# Sclerosing Angiomatoid Nodular Transformation of Spleen (SANTS); Case Report of a 12-Year-Old Patient

**DOI:** 10.30699/ijp.2025.2034980.3332

**Published:** 2025-03-10

**Authors:** Farzaneh Ramezani, Sare Kamali, Ramin Mashoufi, Seyed Ali Ebrahimi, Maryam Soltan

**Affiliations:** 1 *Department of Pathology, Faculty of Medicine, Mashhad University of Medical Sciences, Mashhad, Iran*; 2 *Department of Pathology, Faculty of Medicine, Hormozgan University of Medical Sciences, Bandar Abbas, Iran*; 3 *Department of Radiology, Faculty of Medicine, Mashhad University of Medical Sciences, Mashhad, Iran*; 4 *Department of Medical Sciences, Islamic Azad University, Mashhad, Iran.*; 5 *Department of Pathology, Faculty of Medicine, Isfahan University of Medical Sciences, Isfahan, Iran*

**Keywords:** Splenic neoplasms, Sclerosing angiomatoid nodular transformation, Pediatric spleen lesion, Case report

## Abstract

**Background:**

Sclerosing angiomatoid nodular transformation of the spleen (SANT) is a rare, benign vascular lesion predominantly described in adults. Pediatric cases are exceptionally uncommon and present a diagnostic challenge due to nonspecific clinical presentations and imaging findings.

**Case Presentation:**

We report the case of a 12-year-old boy presenting with recurrent abdominal pain localized around the umbilicus, accompanied by intermittent nausea over a three-month period. Physical examination revealed mild tenderness without guarding. Laboratory findings were unremarkable. Abdominal ultrasound demonstrated a hypoechoic splenic lesion, further evaluated by multidetector computed tomography (MDCT), which revealed a heterogeneous hypodense mass in the spleen. The patient underwent partial laparoscopic splenectomy. Histopathological examination showed a nodular architecture with fibrous bands, capillary-like vascular channels lined by endothelial cells, and a lymphoplasmacytic infiltrate. Immunohistochemical staining was positive for CD31, CD34, and CD8, supporting the diagnosis of SANT.

**Conclusion:**

Although benign, SANT can mimic more aggressive splenic pathologies. This case underscores the importance of considering SANT in the differential diagnosis of splenic masses in pediatric patients and highlights the role of histopathology and immunohistochemistry in achieving a definitive diagnosis.

## Introduction

Sclerosing angiomatoid nodular transformation (SANT) of the spleen is a rare, benign vascular lesion first described by Martel et al. in 2004. It is histologically characterized by multiple angiomatoid nodules embedded within a fibrosclerotic stroma and is most commonly found in middle-aged women. The etiology of SANT remains unclear, although it is hypothesized to arise from exaggerated nodular transformation following prior splenic injury or inflammation. Some studies suggest associations with trauma or hamartomas. Additionally, SANT has been linked to Epstein-Barr virus (EBV) infection—supporting its classification as an inflammatory pseudotumor—or immunoglobulin G4-related autoimmune disease.

Pediatric cases of SANT are extremely rare, with only a few cases documented in the literature. These cases are of particular interest due to the diagnostic challenges they present and the potential for confusion with malignant splenic lesions such as lymphoma or metastatic disease.

Herein, we report a rare case of SANT in a 12-year-old boy who presented with recurrent abdominal pain. We highlight the clinical, radiological, surgical, and histopathological features that led to the final diagnosis.

## Case Report

A 12-year-old boy presented to our center with a three-month history of intermittent abdominal pain. The pain was mild, dull in nature, and localized around the umbilicus, with occasional extension to the right and left upper abdominal quadrants. The pain was non-radiating. The patient also reported intermittent episodes of nausea, though he was asymptomatic at the time of examination.

On physical examination, slight abdominal tenderness was noted without guarding or rebound tenderness. Deep palpation of the abdomen did not reveal any masses or areas of increased tenderness. The patient had no notable past medical history and had previously received symptomatic treatment for abdominal pain with no lasting relief.

Laboratory investigations, including a complete blood count, were within normal limits. There was no evidence of anemia, leukocytosis, or platelet abnormalities.

An abdominal ultrasound was performed, which revealed a hypoechoic lesion measuring 47 × 35 mm located in the inferior pole of the spleen. No other abnormalities were detected. While SANT typically appears as a well-circumscribed, hypoechoic or heterogeneous splenic lesion on ultrasound, these features are not unique and do not allow for definitive diagnosis.

Further evaluation with a multidetector computed tomography (MDCT) scan confirmed a normal-sized spleen with a well-defined heterogeneous hypodense lesion measuring 51 mm in the lower pole. The lesion lacked calcification, macroscopic fat, or surrounding inflammatory changes. The radiological impression was that of a nonspecific splenic mass. The differential diagnosis included hemangioma, hamartoma, fibroma, or inflammatory pseudotumor, though no specific diagnosis could be established based on imaging alone.

Due to the persistent symptoms and inconclusive imaging, the patient was admitted for surgical evaluation. After completing preoperative laboratory testing, a partial laparoscopic splenectomy was performed. The decision to opt for partial splenectomy was made to preserve immune function in this pediatric patient, while also obtaining tissue for definitive diagnosis and relieving symptoms that had not responded to conservative treatment. Intraoperatively, a well-circumscribed, firm mass approximately 5 cm in diameter was visualized at the inferior pole of the spleen. The mass was excised with a 10 cm margin.

Gross examination of the excised specimen revealed a bulging, well-demarcated mass with a variegated cut surface. The tissue included alternating red hemorrhagic areas and tan-yellow fibrous zones. Histological examination with hematoxylin and eosin staining showed a nodular architecture separated by fibrous bands. The nodules were composed of numerous capillary-like vascular spaces lined by plump endothelial cells set in a fibrotic stroma, with a dense lymphoplasmacytic infiltrate.

Given the histological suspicion for SANT, immunohistochemical staining was performed. The lesion demonstrated a characteristic mixture of vascular structures: sinusoidal vessels that were CD31 and CD8 positive but CD34 negative; capillary-like vessels that were CD31 and CD34 positive but CD8 negative; and small vein-like vessels that were CD31 positive but negative for both CD34 and CD8. This immunophenotypic profile confirmed the diagnosis of sclerosing angiomatoid nodular transformation of the spleen.

The patient had an uneventful postoperative recovery and was discharged home two days after surgery. Follow-up evaluations, including physical examinations and abdominal ultrasounds every three months, have shown no recurrence, and the patient remains symptom-free to date.

**Fig. 1:**
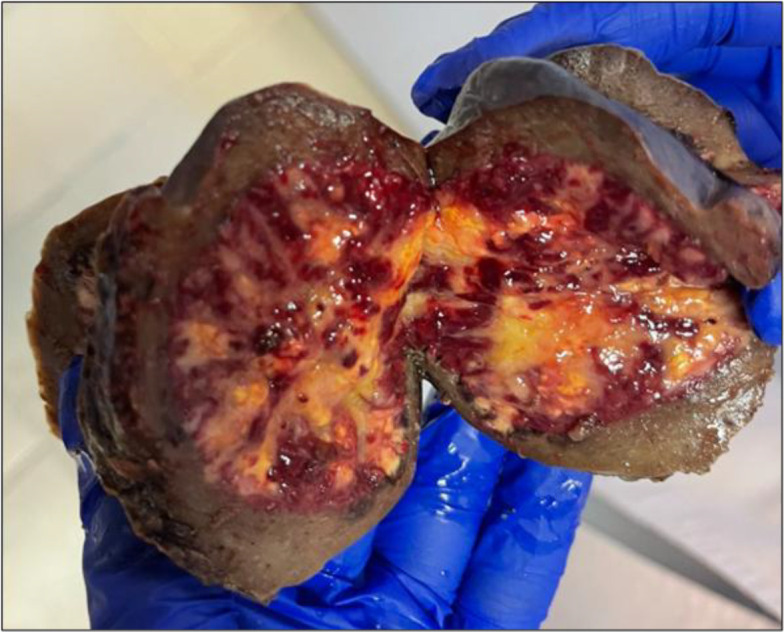
Macroscopic view of the resected splenic lesion showing a well-circumscribed, variegated mass in a pediatric patient.

**Fig. 2:**
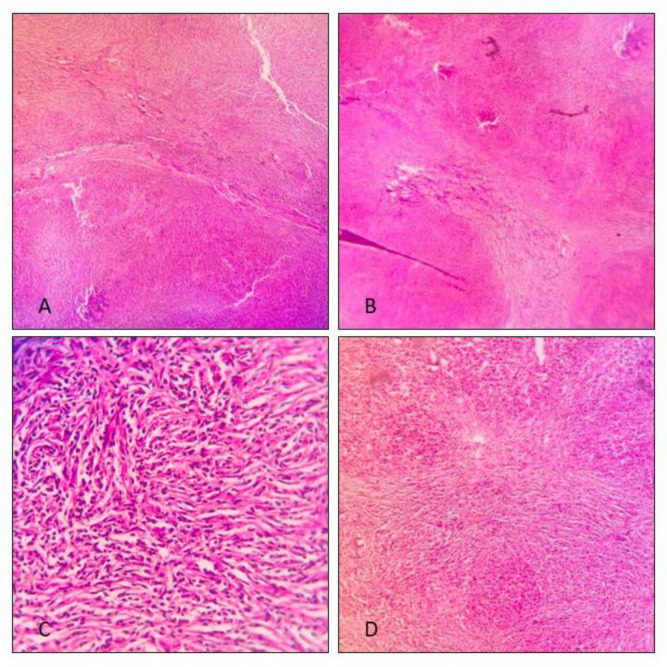
Hematoxylin and eosin stain microscopic examination of the lesion. A and B show examination on 40x magnification. Nodular architecture, with fibrous bands delineating paler nodules are visible. C and D show examination on 100x and 400x magnification respectively. Capillary-like vascular spaces lined by plump endothelial cells in a background of fibroblastic stroma and lymphoplasmacytic infiltrate is visible.

## Discussion

Sclerosing angiomatoid nodular transformation of the spleen (SANT), though well-documented in adults, remains a rare and atypical entity in pediatric populations. A systematic review reported the average age of SANT diagnosis as 45.9 years, with a standard deviation of 15 years. Thus, the case of a 12-year-old boy with recurrent abdominal pain and a splenic mass highlights both the rarity of pediatric presentations and the diagnostic challenges they present. It underscores the importance of including SANT in the differential diagnosis of splenic lesions in children.

The patient's nonspecific symptoms—abdominal pain localized around the umbilicus and intermittent nausea—are consistent with prior SANT reports, where symptoms typically arise due to the mass effect rather than systemic manifestations. The absence of notable physical examination findings, apart from mild tenderness, makes early clinical suspicion of SANT difficult. Moreover, normal laboratory parameters, including complete blood count, are commonly observed in SANT, as the lesion typically does not cause hematologic abnormalities. However, rare reports have described hematologic complications associated with SANT, including anemia or thrombocytopenia.

Radiologically, the findings in this case aligned with those described in the literature. The ultrasound revealed a hypoechoic lesion, a feature present in approximately 88% of previously reported cases. On computed tomography (CT), the lesion was heterogeneous and hypodense, which corresponds to findings observed in about 92% of cases. Although the characteristic “spoke-wheel” enhancement pattern associated with SANT was not mentioned in this case, its absence does not exclude the diagnosis, as this imaging feature is not universally observed. Due to the lack of pathognomonic radiological signs, the lesion was initially suspected to be a hemangioma, hamartoma, fibroma, or inflammatory pseudotumor, reflecting the nonspecific nature of SANT on imaging. In pediatric populations, the differential diagnosis should also strongly consider lymphoma. However, typical imaging characteristics of lymphoma—such as splenomegaly, poorly defined lesion margins, and minimal contrast enhancement—were not present in this case.

Despite advances in imaging, a definitive diagnosis of SANT requires histopathological confirmation due to overlap with other splenic pathologies. The decision to proceed with partial laparoscopic splenectomy in this patient was based on the need for a definitive diagnosis, symptom control, and spleen preservation. Macroscopically, the lesion appeared as a well-circumscribed, multinodular mass with regions of hemorrhage and fibrosis, which is typical of SANT. Histologically, the lesion displayed the classic pattern of multiple angiomatoid nodules separated by dense fibrosclerotic stroma. The variable cellularity and vascular arrangement distinguish SANT from other vascular tumors or inflammatory conditions of the spleen. These histological characteristics are critical for differentiating SANT from conditions such as splenic hemangioma, lymphangioma, and inflammatory pseudotumor.

Immunohistochemical (IHC) staining played a central role in establishing the diagnosis. The lesion was positive for CD31, CD34, and CD8, with three distinct vascular components corresponding to normal splenic red pulp: cord capillaries expressing CD34 and CD31 but lacking CD8, splenic sinusoids positive for CD31 and CD8 but negative for CD34, and small vein-like vessels that expressed CD31 only. This tri-phasic vascular pattern is a hallmark of SANT. The lack of D2-40 staining in endothelial cells further confirmed the vascular, rather than lymphatic, origin of the lesion.

Differentiation of SANT from other splenic lesions is supported by unique IHC profiles. For example, littoral cell angioma expresses CD68 and CD21 but not CD34 or CD8; splenic hemangiomas are CD31 and CD34 positive but usually negative for CD8, CD21, and CD68; lymphangiomas stain positive for D2-40 and negative for CD21 and CD8; splenic hamartomas are positive for factor VIII, CD31, CD8, and type IV collagen, but negative for CD21 and CD68; while angiosarcomas are distinguished by cytologic atypia, high mitotic activity, and partial expression of endothelial markers such as CD31 and CD68. These distinctive staining patterns are essential in ensuring accurate diagnosis and appropriate management.

The treatment of SANT often involves splenectomy, largely due to its resemblance to malignant splenic tumors on imaging. Recent literature, however, suggests that for asymptomatic cases with characteristic radiologic features, conservative management may be an appropriate alternative. A 2020 systematic review by Eede et al. proposed that core needle biopsy, when safely feasible, could confirm the diagnosis and allow for observation in selected patients. However, this approach has not been validated in prospective clinical trials. Given the patient’s symptomatic presentation, young age, and the need for diagnostic certainty, surgical intervention in this case was justified.

Of particular note is the choice of laparoscopic partial splenectomy. While most previously reported SANT cases underwent open total splenectomy, recent cases have demonstrated the feasibility of laparoscopic approaches. Partial splenectomy offers the additional advantage of preserving splenic immune function, which is especially important in pediatric patients. In this case, the lesion's location in the inferior pole allowed for complete excision with adequate margins while sparing the rest of the spleen.

The patient recovered uneventfully and remains symptom-free during follow-up. Although SANT is considered a benign lesion, with no reported cases of malignant transformation, recurrence, or metastasis, long-term follow-up remains prudent, particularly in pediatric patients where long-term outcomes are less well studied.

## Conclusion

This case underscores the importance of including SANT in the differential diagnosis of splenic masses, even in atypical demographics such as pediatric patients. Although imaging may suggest the diagnosis, SANT remains primarily a histopathological diagnosis, emphasizing the critical role of the pathologist in confirming its presence. Despite its benign nature, SANT can mimic more aggressive splenic pathologies, making surgical intervention both diagnostic and therapeutic.

The successful application of laparoscopic partial splenectomy in this case demonstrates that organ-preserving approaches are feasible and beneficial in selected patients, particularly in the pediatric population where immune function preservation is paramount. However, there remain significant gaps in knowledge regarding the pediatric presentation, natural history, and optimal diagnostic strategies for SANT.

Long-term follow-up is essential to monitor for recurrence and to further characterize the clinical course of SANT in children. Additional case reports and larger studies are needed to enhance our understanding and to guide evidence-based management of this rare splenic lesion in the pediatric population.
